# Electro-Acoustic Behavior of the Mitotic Spindle: A Semi-Classical Coarse-Grained Model

**DOI:** 10.1371/journal.pone.0086501

**Published:** 2014-01-30

**Authors:** Daniel Havelka, Ondřej Kučera, Marco A. Deriu, Michal Cifra

**Affiliations:** 1 Institute of Photonics and Electronics, Academy of Sciences of the Czech Republic, Prague, Czechia; 2 Department of Electromagnetic Field, Faculty of Electrical Engineering, Czech Technical University in Prague, Prague, Czechia; 3 Institute of Computer Integrated Manufacturing for Sustainable Innovation, Department of Innovative Technologies, University of Applied Sciences and Arts of Southern Switzerland (SUPSI), Manno, Switzerland; Florida State University, United States of America

## Abstract

The regulation of chromosome separation during mitosis is not fully understood yet. Microtubules forming mitotic spindles are targets of treatment strategies which are aimed at (i) the triggering of the apoptosis or (ii) the interruption of uncontrolled cell division. Despite these facts, only few physical models relating to the dynamics of mitotic spindles exist up to now. In this paper, we present the first electromechanical model which enables calculation of the electromagnetic field coupled to acoustic vibrations of the mitotic spindle. This electromagnetic field originates from the electrical polarity of microtubules which form the mitotic spindle. The model is based on the approximation of resonantly vibrating microtubules by a network of oscillating electric dipoles. Our computational results predict the existence of a rapidly changing electric field which is generated by either driven or endogenous vibrations of the mitotic spindle. For certain values of parameters, the intensity of the electric field and its gradient reach values which may exert a not-inconsiderable force on chromosomes which are aligned in the spindle midzone. Our model may describe possible mechanisms of the effects of ultra-short electrical and mechanical pulses on dividing cells—a strategy used in novel methods for cancer treatment.

## Introduction

Mitosis is a crucial step in cell division of eukaryotic cells, *i.e.* those forming animals, plants, fungi, and protists. During mitosis a cell separates its duplicated genetic information encoded in its chromosomes into two identical ensembles. Errors in this process may lead to genetic disorders and are also supposed to play a role in cancer and aging [1], [2]. The main part of the apparatus involved in the mitosis process is formed by microtubules, tubular cytoskeletal polymers, arranged in a so-called mitotic spindle.

Microtubules (MTs in following) are most frequently formed by 13 protofilaments organized in a hollow cylinder. Each protofilament comprises of a chain of alternating alpha- and beta-tubulin monomers. MTs can be found either stable or highly dynamic. Dynamic instability of MTs resides in rapid switching between growth and shrinkage. These MTs are structurally polar and the charge bound in their structure (see models in refs. [3]–[5]) is also responsible for their electrical polarity, which is generally supposed to play an important role in the function of proteins [6]. MTs can be moved and oriented both in static and alternating electric fields as a result of electrophoresis and dielectrophoresis [7]–[12]. Also, it has been proved that a high magnetic field effects the alignment of MTs [13]–[15], due to diamagnetic anisotropy. Experimental data about mechanical properties of MTs vary over several orders of magnitude [16]. Dynamic mechanical properties of MTs have been studied only on a theoretical level [17]–[30]. MTs should be able to vibrate at their natural frequency according to these studies. In combination with their electrical properties, basic electrodynamic models of MTs have been recently introduced [31]–[34]. These models have enabled the calculation of spatial distribution and time evolution of high-frequency electric fields generated by the single vibration mode of microtubule and MT networks. It has been predicted that MTs should exhibit such electrodynamic activity *in vivo*. The importance of this activity resides in the fact that it (i) may provide an intracellular signaling mechanism [35], (ii) may locally change the chemical reaction rates by attracting or rotating the molecular reaction partners [36].

Microtubules assemble mitotic spindles in a way resembling field lines of an electric dipole. MTs grow from one microtubule organizing center (MTOC, the pole) towards the other. In the spindle midzone (equatorial plane), where chromosomes are arranged, the MTs are either linked to each other (polar microtubules) or bounded to chromosomes (kinetochore microtubules). MTs which ray from MTOC and do not grow towards the equatorial plane are also present (astral microtubules). The mitotic spindle is a dynamic structure which undergoes permanent remodeling in order to capture and segregate chromosomes between daughter cells.

Due to their polarity and involvement in mitosis (and also in other crucial physiological processes), MTs are often targets of research connected to chemical and physical treatment of various diseases. In general, medical strategies aimed at microtubules which form the mitotic spindle are useful in those cases when the rapid rate of cell division is to be interrupted or when an apoptosis triggered by a disruption of the mitotic spindle is to be initiated. Chemical anti-microtubule agents, which cause the aggregation of tubulin or dissociation of MTs, are therefore in widespread clinical use against various types of cancer [37]. Thanks to the electrical polarity of microtubule subunits, it was shown that tumor growth may be inhibited by alternating the electric field which disrupts proper formation of mitotic spindles in cells [38]. While the effect of the external electromagnetic field of 94 GHz on microtubule dynamics within cells seemed to be purely due to heating [39], other authors found small but significant effects on dividing cells which were exposed to 935 MHz which they interpreted as effects of MT polymerization [40]. These examples are, however, not efficient enough or they have drastic side effects. Better understanding of how the formation and function of the mitotic spindle is controlled is, therefore, important for a general biophysical insight as well as for the development of novel diagnostic methods and therapeutic strategies.

Despite this importance, only a few models addressing the dynamics and physical properties of the mitotic spindle exist. The importance of motor proteins for the assembly of the mitotic spindle was analyzed in ref. [41]. The biochemical model addressing control pathways of Mitotic Spindle Assembly Checkpoint was also published [42]. The relation between mechano-chemical factors and their contribution to the generation of forces within the mitotic spindle was reviewed in [43]. Authors of ref. [44] developed the physical theory of mechanical second-scale oscillations of the mitotic spindle which takes place during asymmetric cell division and which is attributed to force imbalances produced by assemblies of molecular motors. Mechanical oscillations, with no reference to electrical, with respect to interruption of mitosis by the ultrasound were also subjects of the rough model [45], which predicts the effective range of frequencies to produce such effect as tens of kHz. Electrical oscillations of the mitotic spindle were considered as a speculative mechanism controlling the chromosome separation [46]. However, no qualitative analysis of this phenomenon was shown. In this context, the study of the electric field coupled to mechanical oscillations of mitotic spindles may comprise one of the missing links in comprehension of the dynamic functionality of mitotic spindles (Preliminary conference report on this topic [33] does not cover all necessary features. Furthermore, it considers only optical branch of vibrations of mitotic spindle, which is very special case selected for convenience of calculation in this [33] pioneering study. Moreover, no analysis is provided.).

In this research paper, we present an electromechanical model of mitotic spindle vibrations based on the Microtubule Resonance Dipole Network Approximation (MRDNA) [34]. It estimates the generation of an electromagnetic field and its force effects as a result of either endogenous or forced vibrations of mitotic spindles. This model has relevance for the assessment of

- influence of external electromagnetic and mechanical stimuli on cell division, especially on the process of chromosome segregation,
- role of the hypothetical endogenous electrodynamic phenomena in mitosis regulation and control,
- possible role of electric fields in cancer treatment, and
- general assessment of the dynamic properties of the mitotic spindle.

We report on how a pulsed electric field, which is used for therapeutic strategies against cancer [47] and is common to contemporary electronic devices, may influence the electric field distribution within the equatorial plane where chromosome separation takes place. Our results also clarify the relevance of the endogenous vibrations for regulatory mechanisms in cells. We provide experimentally testable predictions of spectroscopic data for the purpose of validation of the model. Generally, our model provides a framework for the evaluation of driven or endogenous electro-acoustic behavior of the mitotic spindle.

## Results

**Figure 1:**
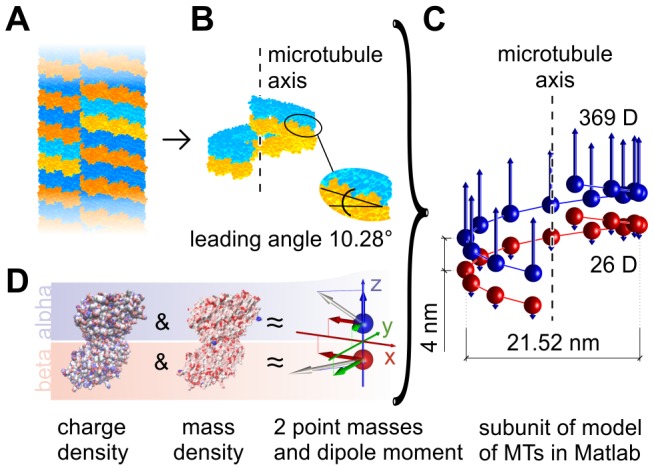
Method of approximation of electric properties of microtubules. The MT (A) is divided into so called MT rings (B) forming the spiral, where corresponding dipoles were placed in the center of gravity of respective monomer (C). Center of gravity and dipoles were calculated from molecular structure of tubulin (D).

We performed computational predictions of the intensity of the electric field generated by acoustic vibrations of the mitotic spindle. In our model, we started with a molecular model of a microtubule (Fig. 1–A), from which we extracted the positions of atoms which form one heterodimer. Since one heterodimer of tubulin contains about 9,000 atoms and the mitotic spindle includes hundreds of thousands of heterodimers, computational demands allow only coarse-grained modeling of the mitotic spindle dynamics. From different combinations of atomic weights and positions we calculated the position of the center of gravity of each monomer of tubulin. We positioned an electric dipole which corresponds to the dipole moment of charge distribution within the monomer into the gravity center of each monomer (Fig. 1–D). Next, we calculated the spatial positions of MTs which form the mitotic spindle and within them coordinates of the centers of gravity for each monomer. Finally, we let the elementary dipoles, representing the electrical polarity of heterodimers, oscillate according to the first natural longitudinal vibrational mode of corresponding MT [23]. We considered two boundary conditions: (i) both ends of all MTs are fixed and (ii) all astral MTs are fixed while equatorial ends of polar and kinetochore MTs are free. We considered two situations in our calculations: (i) synchronized vibrations of the mitotic spindle after pulsed excitation and (ii) vibrations with a random phase and a constant amplitude. The first may come from an externally pulsed electromagnetic or mechanical signal (Note that the pulse duration is bounded with its frequency content. The shorter the pulse, the broader the frequency spectrum.), the latter corresponds to the accumulation of noise and vibrational energy from the inside of a cell [34]. We studied the time evolution of the intensity of the electric field generated by damped and undamped mechanical vibrations of the mitotic spindle for both situations. The notation in figures is as follows: P stands for pulsed excitation; R – random excitation; Fx – fixed-ends; Fe –– free-ends; U – undamped vibrations; D – damped vibrations. For example, PFxU denotes undamped vibrations of MTs with fixed-ends driven by pulse.

**Figure 2:**
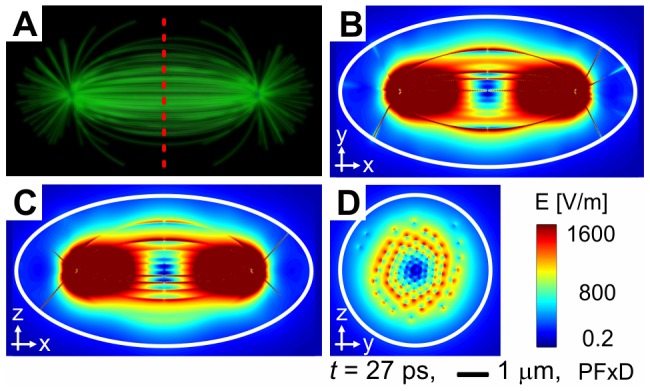
Model of mitotic spindles and corresponding intensities of electric field. The model of mitotic spindle of ellipsoidal cell with equivalent radius 3.3 µm (A). The position of equatorial plane is marked by red dashed line. Magnitude of intensity of electric field in three main plains of the ellipsoidal cell (boundary of the cell is depicted by white circle) in time 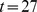 ps after pulsed excitation is shown in (B–D). The plane shown in (D) is the equatorial plane. All pictures have the same spatial and intensity scale and the same quality factor of vibrations, 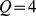. All time locks were taken in 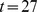 ps after pulsed excitation. Presented data correspond to boundary conditions with fixed ends.

We used ellipsoidal cell models. We positioned 300 microtubules within each cell: 100 astral, 100 kinetochore and 100 polar microtubules. We calculated detailed time and space evolution of the intensity of the electric field for cells with volume equivalent to a sphere with a radius of 3.3 µm (Fig. 2). The equivalent sphere may be understood as the non-dividing version of the ellipsoidal cell. This size was chosen for two reasons. Firstly, such cell has very high microtubule density, so this size of the model may act as an upper estimate of the intensity of electric field in cells. Secondly, small size of the cell reduces the computational demands while sufficient resolution is preserved. We also calculated the spectroscopic properties of this model and of cells with a radius equivalent to 7, 30 and 65 µm to show how the spectrum of vibrations depends on the cell size. The summary of values of specific parameters used in our calculations, as well as mathematical details of the model, are provided in the Models section.

### Spectroscopic properties

**Figure 3:**
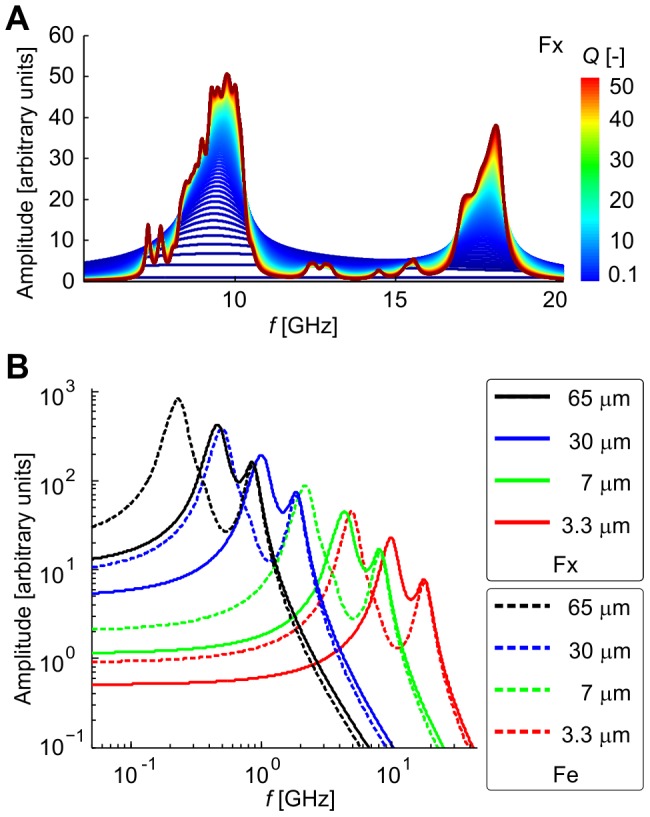
Spectrum of vibrations of mitotic spindle. Spectrum as a function of the quality factor (A) and size of model cell (B). The value of quality factor is coded according to the colorbar in A. Solid line in B corresponds to boundary conditions with fixed ends, dashed line to free ends in equatorial plane.

The essential parameter of oscillatory behavior of MTs is the quality factor of oscillations, which shows how the oscillations are damped. There have been theoretical discussions whether any microtubule vibration modes can be underdamped and thus sustain coherence [48], [49]. Theoretical analysis [30] of forced bending-mode vibrations of MTs in a viscous medium like cytosol revealed that the quality factor of vibrations is frequency dependent and may range from 0 to almost 4. However, there are no experimental works which would quantify the damping coefficient or the quality factor and resolve this discussion. We used values of the quality factor ranging between 0.1 and 50.0 within the parametric space of our model. Values lower than 0.5 correspond to an overdamped system where the responses to impulse driving show no oscillatory behavior but rather exponential decay. We restricted the lower boundary to 0.1 since such a dynamic system becomes too slow and our model might not be appropriate. The upper boundary of 50 reflects the fact that we do not expect microtubule oscillations with higher quality to take place *in vivo*. If it was not so, the oscillations would accumulate a rather large amount of energy which might cause destruction of the system. The upper limit of 50 is higher than that reported in [30] because we consider longitudinal vibration modes in our model. Longitudinal modes are expected to be less damped compared to bending modes since they cause only limited displacement of the surrounding cytosol [31], [50]. The value of the quality factor influences the shape of the frequency spectrum of oscillations: the lower the quality factor, the broader the bandwidth of each MT. The resulting spectrum of the mitotic spindle (see Fig. 3–A) is therefore smooth and broad for smaller values of the quality factor and it shows distinct narrow spectral lines only for higher values of the quality factor. The shape of the spectrum comprises of two bands: the lower one corresponding to relatively longer kinetochore and polar MTs and the higher one corresponding to astral MTs, which are relatively shorter in the considered model. In conclusion, the spectrum of vibrations resembles a histogram of lengths of microtubules with the number of bins proportional to the quality factor.

The bandwidth of the vibrations is given by modes excited in individual MTs. We considered only the lowest longitudinal mode for which we may expect the highest coupling of the driving electric field. The boundary conditions limit the number of half-waves on MT and thus the frequency content. MTs with fixed ends vibrate with a higher frequency than those with one end free. The spectra of the models with different boundary conditions are therefore also different. The other parameter which influences the bandwidth of the spectrum is the size of the model because with increasing size of the spindle the corresponding frequencies become lower. For the largest considered model cell the maximal response occurs at a frequency of approx. 1 GHz while for the smallest cell the spectrum covers a range between approx. 8 and 18 GHz. The shape of the spectrum remains similar and appears to be relatively wider, although the ratio of bandwidth to the central frequency remains unaffected (see Fig. 3–B).

The amplitude of oscillations was set to 1 nm in our simulation, which roughly corresponds to the amplitude predicted by molecular modeling [23]. It means that the maximal longitudinal displacement of the center of gravity of one monomer in the position of the vibration anti-node was 1 nm. Displacements of other monomers within the MT was lower according to the shape of the mode.

### Intensity of the electric field

The high-frequency electric field generated by the vibrations of the mitotic spindle has a very complex structure with high intensity fixed maxima around MTs and other moving local minima and maxima, which are given by destructive and constructive interferences of contributions from all MTs. Generally, the shape of the field changes very rapidly within few nanoseconds as a results of high frequencies involved in vibrations. The intensities of the field vary on very small distances within few orders of magnitude, which leads to gradients as large as 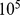 V/m on the distance of few hundred nanometers. The field either pulsates if the vibrations are synchronized to some extent, or has a rather runny character if the phases of vibrations are highly desynchronized. Videos of time evolution of the field are provided in the supplementary material.

Since the most important activity of the mitotic spindle takes place in the spindle midzone where chromosomes are aligned, we analyzed mainly the intensities of the field in the equatorial plane of our model cell, which corresponds to the spindle midzone. The model of the mitotic spindle and examples of time-locks of intensity of the electric field in equatorial and other main plains of the ellipsoid forming the model cell are shown in Fig. 2. The electrical intensity is coded according to the provided scale-bar. In order to show the influence of the chosen parameters of calculations on the resulting intensity, we analyzed the behavior of the model for different boundary conditions and the feeding of vibrations.

It is clearly visible in Fig. 2 that the magnitude of intensity of the electric field has the highest values in the immediate vicinity of MTs. Therefore, the smaller model cells with denser MT networks show higher average intensities of electric fields (data not shown). Very high intensities of electric field are around the MTOCs for the same reason.

**Figure 4:**
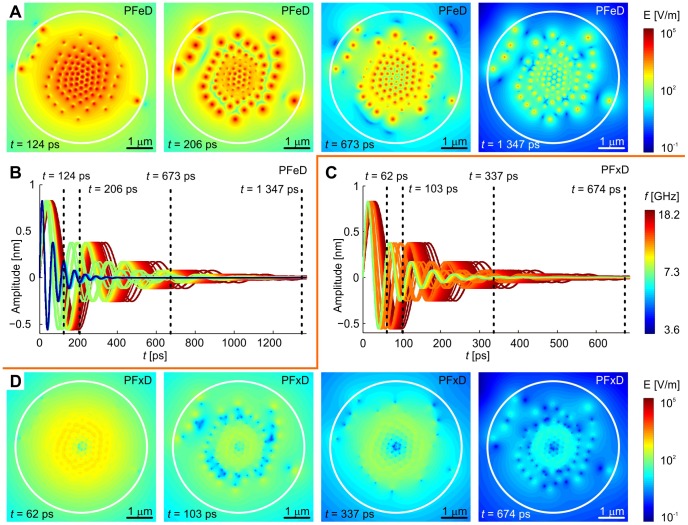
Time evolution of intensity of electric field in equatorial plane for different boundary conditions. The magnitude of intensity of electric field depends on time and boundary conditions. It undergoes exponential decay due to damping after pulsed excitation. Map of electrical intensities (A) and amplitude of mechanical oscillations of all MTs (B) are shown for free ends of MTs in equatorial plane. Data for fixed boundary conditions are shown in (C) and (D). Data correspond to ellipsoidal cell with equivalent radius of 3.3 m. Note different time scales in (A, B) and (C, D). Quality factor of vibrations, 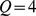, is the same for all examples.

Due to synchronization, pulse-driven oscillations generate higher intensity electric fields at the beginning. This synchronization is lost in the course of time due to different lengths and therefore different frequencies of individual MTs. The oscillatory behavior of the resulting intensity therefore vanishes after few periods. The amplitude of intensity decreases due to damping and to lesser extent also due to desynchronization (*i.e.* lower probability of constructive interferences). It is clearly visible how oscillations driven by pulse die out within 1 ns (Fig. 4). The time scale of vibrations, which is given by their frequencies, therefore leads to events much faster than the dynamic functionality which is visible in microscopic experiments.

Free ends of MTs generate higher electrical intensity in the equatorial planes of cells. The reason for this behavior resides in the fact that there is higher displacement of dipoles in free ends of MTs within the equatorial plane. The higher the displacement, the higher the generated intensity of the electric field. Compared to fixed-end conditions, free ends generate an electric field which is stronger by a few orders of magnitude (Fig. 4).

**Figure 5:**
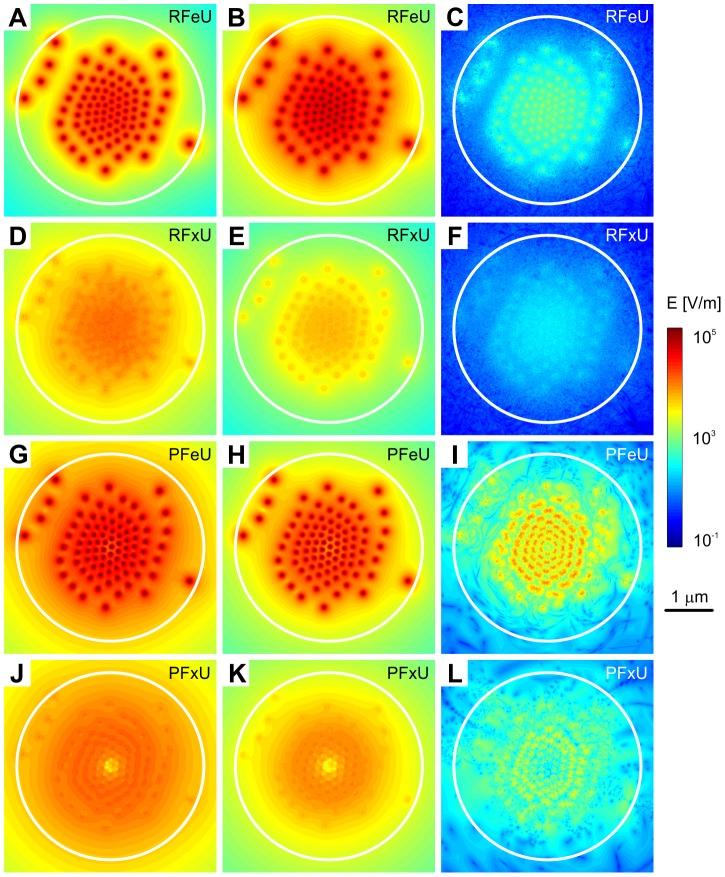
Statistical analysis of electric field generated by random and fixed undamped vibrations. Data are shown for free-ends boundary conditions (A–C, G–I) and for fixed-ends in equatorial plane (D–F, J–L). Maximal values (left column A, D, G, J), mean values (middle column B, E, H, K) and minimum values (right column C, F, I, L) of the intensity of electric field in equatorial plane are shown. Data corresponding to random vibrations are shown in (A–F), results for pulse-driven vibrations are displayed in (G–L).

**Figure 6:**
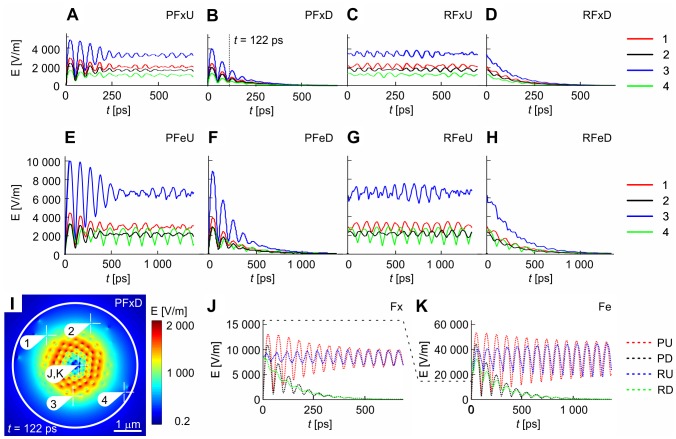
Time evolution of intensity of electric field in equatorial plane for different feeding. Time versus electric intensity plots are shown for fixed-ends and pulsed undamped (A), pulsed damped (B), random undamped (C) and random damped (D) excitation followed by free-ends and pulsed undamped (E), pulsed damped (F), random undamped (G) and random damped (H) excitation shown in points 1–4 of equatorial plane specified in time-lock (I). (J and K) show time versus electric intensity plots in central point of the equatorial plane. Fixed-end boundary condition is displayed in (J), free-end boundary condition is displayed in (K). Dashed curves show data for pulsed undamped, pulsed damped, random undamped and random damped conditions.

Endogenous undamped vibrations with random phases create more unstable structures of the field which, however, looks similar to the previous one in its time-locks. The time evolution does not show any regularity since interferences occur randomly. We therefore analyzed the statistical distribution of the intensity of the electric field in time and space using the Monte Carlo approach, *i.e.* by statistical analysis of many simulations with random initial conditions. The maximum, minimum and mean values of electrical intensity within the equatorial plane are shown in Fig. 5 (A–F). Data were collected from 5 periods of vibrations of the longest MT for 100 random initial conditions. 300 samples were statistically analyzed from each realization. Again, free-end boundary conditions lead to much higher electrical intensities in the equatorial plane compared to fixed-end boundary conditions. Despite no distinctive regularity in terms of the field shape, there was only a little variation from run to run in terms of average intensity of electric field. The same type of data calculated for the pulse driven vibrations are shown in Fig. 5 (G–L) for comparison. The differences in the time evolution of the electric field in the equatorial plane between pulsed and random excitation is illustrated in Fig. 6. The pulsed excitation leads to higher amplitudes of electric intensity, while the mean value at the point of evaluation is almost identical for both feedings due to constructive interference.

In summary, the maximal intensities of the electric field are achieved (i) in the vicinity of MTs (ii) in the area of the highest amplitude of vibrational displacement and (iii) shortly after excitation due to maximum displacement of dipoles. We may generally conclude that the absolutely highest magnitude of intensity of the electric field within the equatorial plane is achieved after synchronized excitation in cells with high density of the MT network, where MTs are not fixed in the midzone. However, the mean value of the intensity does not depend on the type of excitation.

## Discussion

This is the first documented report of a model concerning the electro-acoustic behavior of the mitotic spindle. This model provides valuable data for future research of mitotic spindle dynamics. It also provides foundations for assessment of the role of large scale bio-electrodynamic phenomena in cell physiology. In the following section, we discuss the limitations of our model, the selection process of used parameters, physical and biological relevance of results and possibilities of verification of the model and its computational predictions.

### Limitations of the model

The model provides the very first insight into electro-acoustic behavior of the mitotic spindle. In this section, we will analyze the limitations of the model and discuss possible biological and biophysical implications of the results.

The limitations of the model are caused by simplifications which were necessary for efficient calculation. First of all, the real atomic arrangement of MTs was replaced with a coarse-grained representation where each monomer was approximated with its mass and a dipole moment. Such an approximation leads to loss of details of the consequently generated field on the scales which is comparable to the size of one monomer. However, since the field for each point in space is a summation of the total contribution from all dipoles, the loss of details is not so dramatic. Moreover, it may be considered as an advantage because it provides averaged values which may allow more general conclusions (they are less sensitive to the nanometer-detailed spatial arrangement of the mitotic spindle). A more detailed structure of the field would probably not have such an effect on the chromosomes with dimensions much larger than one tubulin heterodimer.

The second limitation resides in the fact that our model is based on the assumption that there is no back-coupling of the oscillating field to mechanical oscillations. We allowed ourselves to neglect any back- coupling because we suppose that mechanical oscillations of a single MT already take the coupling into account since mechanical properties are, besides other forces, given by the electromagnetic interaction between atoms constituting the MT. The interaction between MTs was neglected because of computational demands. From the results we may say that it does not affect the resulting field in the equatorial plane because MTs are rather far from each other with respect to the intensity of the field they generate. A different situation is near the MTOC, where the microtubules' network is dense. Coupling in this region should be analyzed in future research.

Thirdly, the oscillations of individual MTs were calculated with boundary conditions independent from oscillations of other MTs. It means that MTOCs were considered absolutely rigid. As a result, the overall vibrations of the MTs in our model probably do not correspond to natural vibrations of the mitotic spindle. However, it is not to the detriment of generality of our results because the parameters of the coupling between MTs forming the mitotic spindle are not known anyway. Also, no boundary conditions can be generally stated.

### Parameters used in calculations

Like in any other model or rather a complicated system, our model of the mitotic spindle has a large number of degrees of freedom, *i.e.* a large number of parameters which we had to choose. Some values of the used parameters are either simply unknown or subject to biological variability.

The size, geometry and number of MTs forming the spindle vary for diverse cells and it is not possible to generalize this variability in one model. However, our model is rather robust in this sense and enables modifications thanks to possible separation of the geometrical model and electromagnetic computations. The vibrational properties are the most speculative part of our model. The damping of vibrations, type of excited modes, excitation itself and boundary conditions may be only estimated. The frequency of the lowest vibration mode of MT was extrapolated from the molecular dynamics and normal model analysis data, the most accurate up to date model available. The selection of the longitudinal type of modes was based on their highest efficiency for generation of an electric field and assumed low damping. Bending modes involve much greater displacement of surrounding mass and therefore are likely much more damped. Torsional modes involve much smaller total electric dipole moments due to cylindrical symmetry and therefore their efficiency of generation and absorption of electric field is likely low. There is only a single report which allows estimates of microtubule vibration damping [51] and yields 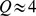 from halfwidth 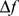 of the spectral peak at the central frequency 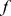 assuming 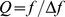. Homogenization of electrical properties of the remaining cells was necessary for obtaining generalized results. Other approximations do not influence the fundamental behavior of the model.

### Discussion of results

Our *in silico* experiments have shown that mechanical vibrations of microtubules which form the mitotic spindle generate an oscillating high frequency electromagnetic field. The relevance of an electromagnetic field in cell biology resides in the force by which it may act on the matter. The electromagnetic field may influence particles which are electrically charged, polarized or polarizable. Since the magnetic component of the field is very low in our simulations (data not shown), we may limit the following discussion to only the effects of the electric component in the field.

The intensity of the electric field has reached the highest magnitude in the surroundings of microtubules' vibration anti-nodes, within the area with high microtubule density and, to lesser extent, also in cases of constructive interference of contributions from more MTs. The position of the regions of high intensity of the field is therefore dependent on the boundary conditions of microtubules' vibrations. The question whether MTs have a vibration node or anti-node in their equatorial plane is therefore important for further evaluation of possible effects of the generated field. We may expect that the free-end condition (anti-node in the equatorial plane) is closer to reality because the MTs in the spindle midzone are either free searching for chromosomes (kinetochore MTs) or bound to each other by motor proteins (polar MTs). The binding of MTs to motor proteins can not be definitely represented by only free MT ends, but we may expect that the binding is either (i) stiff or (ii) pliant. In the first case, the vibrations will be effectively coupled between MTs and, therefore, there will not be a single node in the spindle midzone. In the second case, the weak coupling between MTs will most likely lead to the formation of a local anti-node. We may, therefore, expect that the intensity of the electric field would be reaching a higher range of values presented in our results. The intensity also depends on the amplitude of mechanical vibrations. We used maximum amplitude in anti-nodes of 1 nm, which is comparable to the amplitude of vibrations measured on single cells [52]. These vibrations have much lower frequency than those discussed here, but such an amplitude may be reached after artificial excitation.

Generally, vibrations excited by pulse generate the same mean intensity as random vibrations, both without damping. The issue of damping is very important in our model but its relevance for random vibrations is questionable, because it is extremely difficult to estimate thermal excitation of the mitotic spindle. The relevance of the presented data is in comparison with pulsed excitation. Since the mean value of generated intensities is the same, the question of phase synchronization was proved to be not of much importance for the evaluation of driven vibrations. It means that external feeding does not need to take into account the space/time/phase properties of vibrations in order to be excited. Only the constructive interference, which has limited effect, will disappear while the main behavior features exhibited by our model will not be affected.

Since the functional role of the mitotic spindle takes place within the spindle midzone, we were mostly interested in the field properties in the equatorial plane of our model cell. The intensities of the field in the equatorial plane were as large as 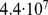 V/m. The intensity may vary within several orders of magnitude in space and time. Differences up to 5 orders of magnitude of V/m may be found on the distance of hundreds of nanometers and even larger differences occur in one point of space within hundreds of picoseconds.

The quantitative analysis of possible force effects of electromagnetic fields presented in this research go beyond the scope of this paper. Briefly, the field with comparable frequencies and intensities as large as those shown in this research was experimentally shown to produce observable translational effect on the free MTs. Since chromosomes are also electrically polarizable [53] and larger in size in two more dimensions than MTs, we may also expect them to be more profoundly susceptible to the reported field due to polarization. The idea that the field exerts translational movements of chromosomes for larger distances is rather far-fetched, but the rotational or small scale translational movement may be possible. Such a small scale movement may, however, have a significant effect on the proper binding of chromosomes to MTs. Whether this effect would be constructive or destructive for proper chromosome separation remains unknown, but we may expect that destructive effects are much more probable, because such a complex field could be driven externally for specific purposes only with extreme technical difficulties. Picosecond time-scale of vibrations leads to ultra-fast events which are much faster than standard dynamics of the mitotic spindle. Biological events with this time-scale are explored only a little.

We see the presented model to have relevance for elucidation of the effects of electric pulses during mitosis, especially for cancer treatment. The effects of short nanosecond pulses, be it of electric or acoustic nature, can be described using our model by pulse excitation of the mitotic spindle vibrations. Such pulse excitation of the mitotic spindle generates strong electric field intensity at the centromere searching ends of MTs. This high intensity may have disrupting influence on (i) the kinetochore-microtubule binding process or on (ii) the chromatid separation with consequence in disrupting mitosis. Cancer cells would be especially sensitive to mitosis disruptions due to their high division rate. A more selective targeting process of microtubule-kinetochore binding or chromatid separation could be developed, once the details of microtubule electro-mechanical coupling and vibrational properties are disclosed by experiments.

Further, our model represents a tool for quantification of the role of endogenous cellular high frequency electric field upon which further theoretical and experimental development can be built. High frequency electric oscillations can mediate force interactions between molecules and supramolecular structures over a longer range than electrostatic interactions [54], [55]. This is because any screening of an electric field by ions gets much less effective for higher frequencies of electric oscillations due to limited ion mobility in cytoplasm [55]. This fact opens a new view on possible endogenous mechanisms for recruitment of molecular partners in cells where diffusion is restricted due to macromolecular crowding. Namely, the fluctuating high frequency electric field of microtubule network could provide the long range searching and steering mechanism needed for microtubule-based kinetochore and centromere location and subsequent chromosome segregation.

### Future directions and conclusions

Our model predicts the existence of a rapidly changing electric field generated by either driven or endogenous vibrations of the mitotic spindle. The existence of this field is subject to experimental verification. Well defined *in vitro* spindle models [56] or well described model cells can be used for electrical spectroscopic measurements which could reveal absorption on frequencies corresponding to lengths of MTs. Direct measurement of the MT generated field is considered extremelly challenging even with currently available nanotechnologies [57], but fluorescent probes sensitive to electric fields [58] may bring some initial insight.The biological relevance of electro-acoustic vibrations of microtubules and the mitotic spindle remains to be experimentally tested.

In conclusion, our model enables the analysis of the effects of electro-acoustic vibrations of the mitotic spindle for the very first time. We believe that all the necessary approximations and simplifications did not have a significant effect on the fundamental outputs of our model. The results of our simulations revealed that the electric field coupled to mechanical vibrations of microtubules forming the mitotic spindle has a very complex spatial and temporal structure. For certain values of our parameters, the intensity of the electric field and its gradient reached values which may have a significant effect when subject to biological conditions. For instance, to exert a force on chromosomes aligned within the spindle midzone or on the microtubules which do not undergo vibrations. The model may be applied in various fields dealing with the effects of electric fields on dividing cells, especially in cancer treatment. Future research should pursue (i) experimental verification of predicted behavior and (ii) a more detailed modeling of the mitotic spindle of real model cells. Our key contribution in this research has been to provide our model for future research, showing the biological relevance of the processes it describes.

## Models

The details of the model are described in this section. All the parameters of the model are first shown as variables. The values of these variables which were chosen for our calculations are then summarized in Table 1. The model was developed in Matlab. The code is freely available upon request.

**Table 1:** List of parameters.

Symbol	Description	Value	Units
**D** *_α_*	dipole moment of *α* monomer	369	D
**D** *_β_*	dipole moment of *β* monomer	26	D
*s*	axial shift between protofilamnets	0.92	nm
*ζ*	diameter of MT rings	10.76	nm
Ξ	leading angle of MT rings	10.28	degrees
*a*	major axis of ellipsoid cell	5.16	µm
*b*	minor axis of ellipsoid cell	2.64	µm
*R*	radius of non-dividing spherical cell	3.3	µm
*V*	volume of spherical cell with radius *R*	0.15	µm^3^
*c*	position of MTOC on the x-axis	2.64	µm
*ρ*	diameter of MTOC	200	nm
*N*	number of MTs	300	-
*N_a_*	number of nucleations centers, astral MTs, one MTOC	50	-
*N_k_*	number of nucleations centers, kinetochore MTs, one MTOC	50	-
*N_p_*	number of nucleations centers, polar MTs, one MTOC	50	-
*κ_a_*	equivalent number of nucleation centers	120	-
Ω*_a_*	spatial angle for division of MTOC, astral MTs	2.8212	sr
*κ_p_*_+*k*_	equivalent number of nucleation centers	225	-
Ω*_p_*_+*k*_	spatial angle for division of MTOC, polar and kinetochore MTs	2.9154	sr
*m_q_*	arbitrary constant	1	-
*u*	index of polar and kinetochore MTs	1, 2,…, 200	
*π*	mathematical constant	3.14159…	-
*n*	index denoting *n^th^* MT	1,2,…,*N*	-
*p_aα_*	oscillating part of dipole moment of *α*-tubulin	(3.8)^−1^	-
*p_aβ_*	oscillating part of dipole moment of *β*-tubulin	(3.8)^−1^	-
*Q*	quality factor	0.5÷100	-
*k*_1_	coefficient of extrapolation	2.5304⋅10^12^	-
*r*	radius of outer wall of MT	12.5	nm
*k*_2_	coefficient of extrapolation	9.0966⋅10^8^	-
*l_TH_*	length of tubulin heterodimer	8	nm

The list of symbols (in the order of appearance) representing variables of the model and their values used for calculations.

### The center of gravity of tubulin monomers

The spatial arrangement of atoms within a molecular model of one heterodimer in polymerized MT was used. Each 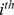 atom within one monomer has its position 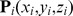 and weight 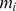. The center of gravity of one monomer was determined as 
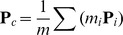
, where 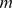 is the total mass of the corresponding monomer.

### Dipole approximation of tubulin monomers

The charge distribution within heterodimers is included in the above mentioned molecular model. The total dipole moment was assessed similarly to the determination of the center of gravity. The charge 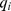 in position 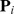 (relatively to the center of gravity) has its dipole moment 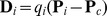. The total dipole moment of one monomer is then given as 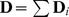 (see Fig. 1).

### MT rings

The parallel arrangement of protofilaments in lattice leads to axial shift 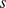 between neighboring protofilaments. The microtubule may be seen as being formed by periodically arranged rings of heterodimers in axial direction. These so called MT rings have the shape of a spiral (see Fig. 1) with a diameter 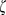 (distance of the center of gravity from the axis) and leading angle of 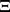 degrees.

### The arrangement of microtubules

**Figure 7:**
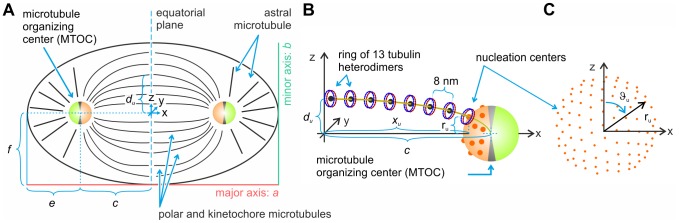
Detailed geometry of the model. The overall structure of mitotic spindle in the model (A). Kinetochore and polar microtubules grow from MTOC along ellipsoidal trajectory towards the equatorial plane (B). The MTOC is devided into two parts. One serves as a base for kinetochore and polar MTs (orange), the second for astral MTs (green). Nucleation centers of MTs are distributed uniformly on the surface of the MTOC (C).

The geometry of the mitotic spindle was calculated in the coordinates shown in Fig. 7–A. The spindle occupies space of an ellipsoid with dimensions 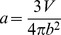 and 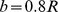, where 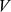 is the volume of a spherical cell with a radius 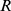 (this radius corresponds to a non-dividing spherical cell with the same volume like a dividing ellipsoidal cell considered here). Microtubule organizing centers (MTOCs) were placed on the x-axis in distance 
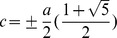
 from the origin of the coordinate system. MTOC, the pole, was represented by a sphere with a diameter 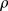 from which MTs grow tangentially. In order to efficiently distribute 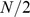 microtubule nucleation centers (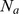 astral, 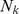 kinetochore and 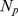 polar microtubules, corresponding to 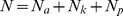 microtubules in our model) on MTOC without any intersections we placed them uniformly according to the following algorithm. We used the equation of symmetrical distribution 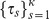 of 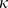 electrically charged particles on a sphere under the condition of the smallest potential energy [59]

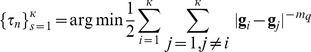
(1)where 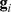 is the position of the 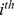 particle and 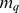 is an arbitrary constant (we used 1). Since we assume that there are more kinetochore and polar MTs than astral MTs, we divided MTOC into two parts given by spatial angles 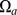 for astral MTs and 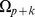 for kinetochore and polar MTs (the corresponding 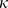 of the model is increased proportionally to the surface of the whole MTOC). Therefore, there are different densities of nucleation centers on each part, so the resulting number of centers of corresponding parts represents 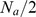 and 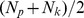 MTs, respectively.

Astral MTs grow from each MTOC radially towards the cell membrane. Kinetochore and polar microtubules grow along the ellipsoidal trajectory which is given by 3 points: the center of MTOC, nucleation center and the magnified projection of nucleation centers to the equatorial plane. The magnification is given by factor
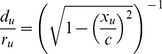
(2)where parameters 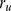 is the distance of the nucleation center of 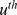 kinetochore or polar MT from the x-axis, 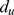 is its magnified projection to the equatorial plane and 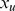 is its x-coordinate (for details see 7–B, C).

### MT ring positions

Once the equations of microtubules' axes were defined, the dipoles representing tubulin monomers were placed along these trajectories. The length of these trajectories, 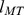, was divided into 8 nm long sections (potential differences were rounded) corresponding to the longitudal size of one heterodimer. MT rings were placed coaxially with the MTs' trajectories at the beginning of each section (see Fig. 7).

### Excitation of vibrations

Finally, the dipole moments of all monomers were modulated both in space and time. These modulations correspond to oscillations of dipoles along the trace of a given vibration mode in the course of time. All dipoles were modulated by the function 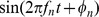, which represents the frequency of vibrations of respective 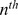 MT (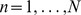), where 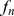 is the frequency of vibration, 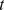 is time and 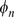 is the phase of 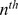 MT. The amplitude and phase of vibrations depends, for each dipole, on its position within the MT. Therefore the dipole moment was also modulated by the function 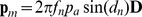, where 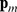 is the modulated dipole moment, 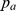 is the oscillating part of the dipole moment of the corresponding monomer (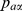 for 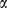-tubulin and 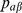 for 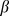-tubulin) and 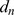 is the amplitude of monomer's displacement given by the wave number.

### Calculation of the intensity of the electric field

**Figure 8:**
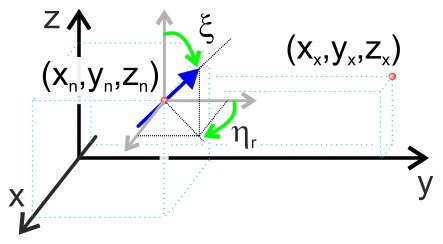
Transformation of coordinates used for calculation of radiation of a dipole. The schematics explains the geometrical meaning of parameters in Eqs. 4–7.

The resulting intensity of the electric field in a point 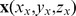 of space was calculated as a vector summation of all the contributions as
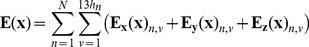
(3)where 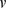 is the index of the electric dipole (tubulin monomer) in 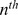 MT, 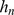 is the number of electric dipoles in one protofilamet of 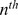 MT (all 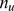 in 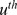 MT are equal), 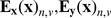 and 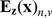 are components of the electrical intensity of electric dipole with indexes 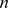 and 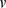 in the direction of respective axes. The relation between 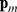 and 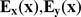 and 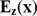 is given by standard equations of dipole radiation. Those equations stand, in Cartesian coordinates and after rotation and translation of the dipole to its proper position, as follows
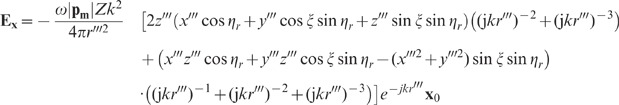
(4)

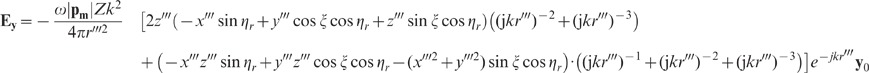
(5)

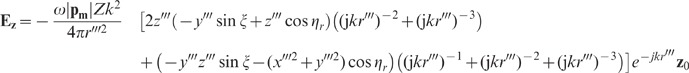
(6)where 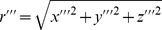, 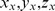 are coordinates of the point of evaluation 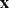. 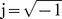 denotes the imaginary constant. 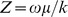 is the impedance of the environment, where 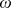 is angular frequency of oscillations, 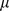 is permeability and 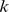 is the wave number. Auxiliary coordinates 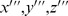, are given as
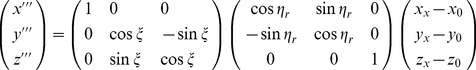
(7)where 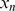, 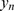 and 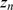 denote axial coordinates of the position of the dipole and 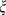 and 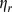 are angles between the dipole and axes z and y, respectively (see Fig. 8).

### Estimation of the spectra of vibrations

The spectra of vibrations were estimated by summation of the Lorentzian curves placed at principal vibrational frequencies of each MT. Assuming each MT to have the principal frequency 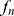 and quality factor 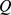, then the resulting spectrum is given by the following equation:
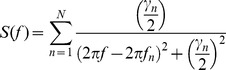
(8)where 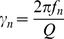, 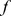 is frequency and 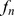 is the resonance frequency of the 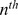 microtubule.

### Parameters of the model

Specific values of parameters of the model are given below.

The molecular model of tubulin was taken from the RCSB Protein Data Bank (1TUB code) and adopted as described in [23]. The resulting model corresponds to structure of heterodimers within polymerized MTs with 13:3 B lattice.

The model cells we used in our calculations have an equivalent radius, 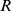, of 65 µm, 30 µm, 7 µm and 3.3 µm. We positioned 300 microtubules within these cells: 100 astral, 100 kinetochore and 100 polar MTs. For positioning of 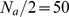 nucleation centers of astral MTs on one MTOC we used 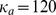 symmetrically distributed points corresponding to a solid angle 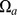 = 2.8212 sr (see Fig. 7–A, green part of MTOC). Correspondingly, we used 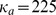 and 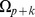 = 2.9154 sr (see Fig. 7, orange part of MTOC) for 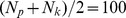 nucleation centers of kinetochore and polar MTs.

**Figure 9:**
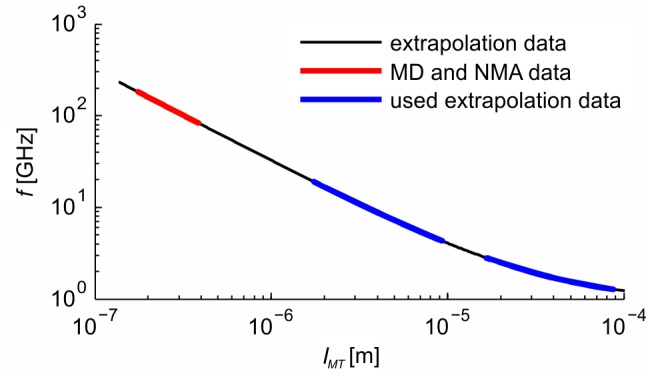
Frequency of vibrations of MT as a function of its length. Molecular dynamics and normal mode analysis models of MT (red) were extrapolated (black) for this purpose to the region of lengths relevant to this model (blue).

The dependency of the resonance frequency on the length of the microtubule was extrapolated from molecular dynamics and normal mode analysis models [23] as
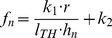
(9)where 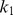 and 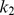 are coefficients of extrapolation (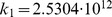 and 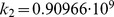), 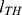 is the length of tubulin heterodimer. This extrapolation (black thin curve) is shown in Fig. 9. The thick red curve was calculated by MD (molecular dynamics) and NMA (normal mode analysis). The frequency range used in our model is depicted by the thick blue line. We used the principal longitudinal vibration mode for excitation of MT. All astral MTs were considered with zero boundary conditions, i.e. with fixed ends on the cell membrane and MTOC. We used two different boundary conditions for kinetochore and polar MTs

1. fixed on both ends of MT and
2. fixed on MTOC and free in equatorial plane.

**Figure 10:**
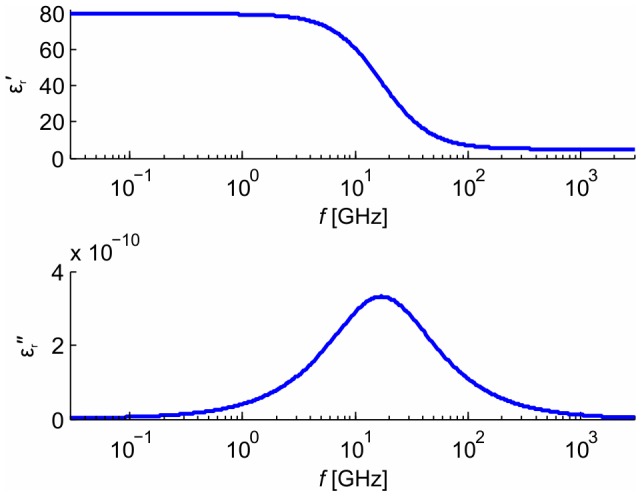
Electrical parameters of the cytosol. We used homogeneous electrical properties of the surroundings of the MTs in our model. The figure shows frequency versus complex permittivity plot. The real part of the complex permittivity (up) represents the value of the relative electrical permittivity, and therefore energy stored in the material, and the imaginary part (down) corresponds to dielectric losses.

The quality factor of vibrations ranging between 0.1 and 50 was used. The maximum amplitude of displacement of the monomer's center of gravity within one MT (in anti-node) was set to 1 nm. The displacements of all other monomers within MT are described by the shape of the vibration mode. The electrical parameters of the surroundings of MTs were considered homogeneous within the volume of the cell. The dielectric permittivity spectrum of the model cytosol is shown in Fig. 10, [60]. All other parameters and their values are summarized in Table 1.

### Resolution

The volume of model cells was segmented into the Cartesian grid of voxels, *i.e.* points of evaluation, with equal dimensions. The edge length of each cubic voxel was 8 nm for cell with equivalent radius of 3.3 µm.

The time resolution in time-evolution simulations was given by the duration of the periods of vibration of MTs. We used time series covering 5 periods of the vibration of longest MT sampled into 300 time steps. This range and sampling together cover a variety of possible interference products, still ensure that the highest vibration frequency is not under-sampled and is computationally affordable.

### The Monte Carlo analysis

The Monte Carlo analysis was used for statistical evaluation of random vibrations. Maximal, mean and minimal value of the intensity of the electric field was estimated in each point of evaluation within the equatorial plane. The analysis was performed for 100 random initial conditions. As is stated above, data were collected from 5 periods of vibrations of the longest MT. 300 equidistant samples were taken from the time of these 5 periods.

## Supporting Information

Video S1 Time evolution of intensity of electric field in equatorial plane for pulsed feeding and free ends.
(AVI)

Video S2 Time evolution of intensity of electric field in equatorial plane for pulsed feeding and fixed ends.
(AVI)

Video S3 Time evolution of intensity of electric field in three major planes of the model cell for pulsed feeding and fixed ends.
(AVI)

Video S4 Time evolution of intensity of electric field in XY plane for pulsed feeding and fixed ends.
(AVI)

Video S5 Time evolution of intensity of electric field in XZ plane for pulsed feeding and fixed ends.
(AVI)

Video S6 Time evolution of intensity of electric field in equatorial plane for random feeding and free ends.
(AVI)

Video S7 Time evolution of intensity of electric field in equatorial plane for random feeding and fixed ends.
(AVI)
