# Correction: Electro-Acoustic Behavior of the Mitotic Spindle: A Semi-Classical Coarse-Grained Model

**DOI:** 10.1371/journal.pone.0210897

**Published:** 2019-01-28

**Authors:** Daniel Havelka, Ondřej Kučera, Marco A. Deriu, Michal Cifra

There are errors in the values reported for parameters *a*, *b*, *c*, and *V* in Table 1. Please see the correct Table 1 here.

**Table 1:** List of parameters.

Symbol	Description	Value	Units
**D**_*α*_	dipole moment of *α* monomer	369	D
**D**_*β*_	dipole moment of *β* monomer	26	D
*s*	axial shift between protofilaments	0.92	nm
*ζ*	diameter of MT rings	10.76	nm
Ξ	leading angle of MT rings	10.28	degrees
*a*	major axis of ellipsoid cell	10.32	μm
*b*	minor axis of ellipsoid cell	5.28	μm
*R*	radius of non-dividing spherical cell	3.3	μm
*V*	volume of spherical cell with radius *R*	150.5	μm^3^
*c*	position of MTOC on the x-axis	3.089	μm
*ρ*	diameter of MTOC	200	nm
*N*	number of MTs	300	-
*N*_*a*_	number of nucleation centers, astral MTs, one MTOC	50	-
*N*_*k*_	number of nucleation centers, kinetochore MTs, one MTOC	50	-
*N*_*p*_	number of nucleation centers, polar MTs, one MTOC	50	-
*κ*_*a*_	equivalent number of nucleation centers	120	-
Ω_*a*_	spatial angle for division of MTOC, astral MTs	2.8212	sr
*κ*_*p*+*k*_	equivalent number of nucleation centers	225	-
Ω_*p*+*k*_	spatial angle for division of MTOC, polar and kinetochore MTs	2.9154	sr
*m*_*q*_	arbitrary constant	1	-
*u*	index of polar and kinetochore MTs	1, 2, …, 200	
*π*	mathematical constant	3.14159	-
*n*	index denoting *n*^*th*^ MT	1,2,…,*N*	-
*p*_*aα*_	Oscillating part of dipole moment of *α*-tubulin	(3.8)^−1^	-
*p*_*aβ*_	oscillating part of dipole moment of *β*-tubulin	(3.8)^−1^	-
*Q*	quality factor	0.5 ÷ 100	-
*k*_1_	coefficient of extrapolation	2.5304 ⋅ 10^12^	-
*r*	radius of outer wall of MT	12.5	nm
*k*_2_	coefficient of extrapolation	9.0966 ⋅ 10^8^	-
*l*_*TH*_	length of tubulin heterodimer	8	nm

The list of symbols (in the order of appearance) representing variables of the model and their values used for calculations.

There is an error in the equation in the third sentence in the “The arrangement of microtubules” subsection of the Models section. The equation describing the distance from the origin of the coordinate system for MTOC placement on the x-axis is incorrect. Please see the correct equation here:

$$c=\pm \frac{a}{1+\sqrt{5}}\mp \frac{\rho }{2}$$

There is an error in the Eq (6) in the “Calculation of the intensity of the electric field” subsection of the Models section. Please see the correct Eq (6) here:

$$E_{z}=-\frac{\omega |p_{m}|Zk^{2}}{4\pi r^{\prime \prime \prime 2}}[2z^{\prime \prime \prime }(-y^{\prime \prime \prime }\;\sin\;\xi +z^{\prime \prime \prime }\;\cos\;\xi )({\left(jkr^{\prime \prime \prime }\right)}^{-2}+{\left(jkr^{\prime \prime \prime }\right)}^{-3})+(-y^{\prime \prime \prime }z^{\prime \prime \prime }\;\sin\;\xi -(x^{\prime \prime \prime 2}+y^{\prime \prime \prime 2})\cos\;\xi )({\left(jkr^{\prime \prime \prime }\right)}^{-1}+{\left(jkr^{\prime \prime \prime }\right)}^{-2}+{\left(jkr^{\prime \prime \prime }\right)}^{-3})]e^{-jkr^{\prime \prime \prime }}z_{0}$$

There is an error in the Eq (7) in the “Calculation of the intensity of the electric field” subsection of the Models section. Please see the correct Eq (7) here:

$$\left(\begin{array}{c} x^{\prime \prime \prime }\\ y^{\prime \prime \prime }\\ z^{\prime \prime \prime } \end{array}\right)=\left(\begin{array}{ccc} 1 & 0 & 0\\ 0 & \cos\xi & -\sin\xi \\ 0 & \sin\xi & \cos\xi \end{array}\right)\left(\begin{array}{ccc} \cos\eta_{r} & -\sin\eta_{r} & 0\\ \sin\eta_{r} & \cos\eta_{r} & 0\\ 0 & 0 & 1 \end{array}\right)\left(\begin{array}{c} x_{x}-x_{0}\\ y_{x}-y_{0}\\ z_{x}-z_{0} \end{array}\right)$$

The authors confirm that the code used in the modelling do not contain the errors in parameters and equations, which affect only the description of the models. The results and conclusions are therefore unaffected by these corrections to the reporting of the methodology.

There are errors in the scale of the y-axis shown for the bottom panel of Fig 10. Please see the correct Fig 10 here.

**Fig 10:**
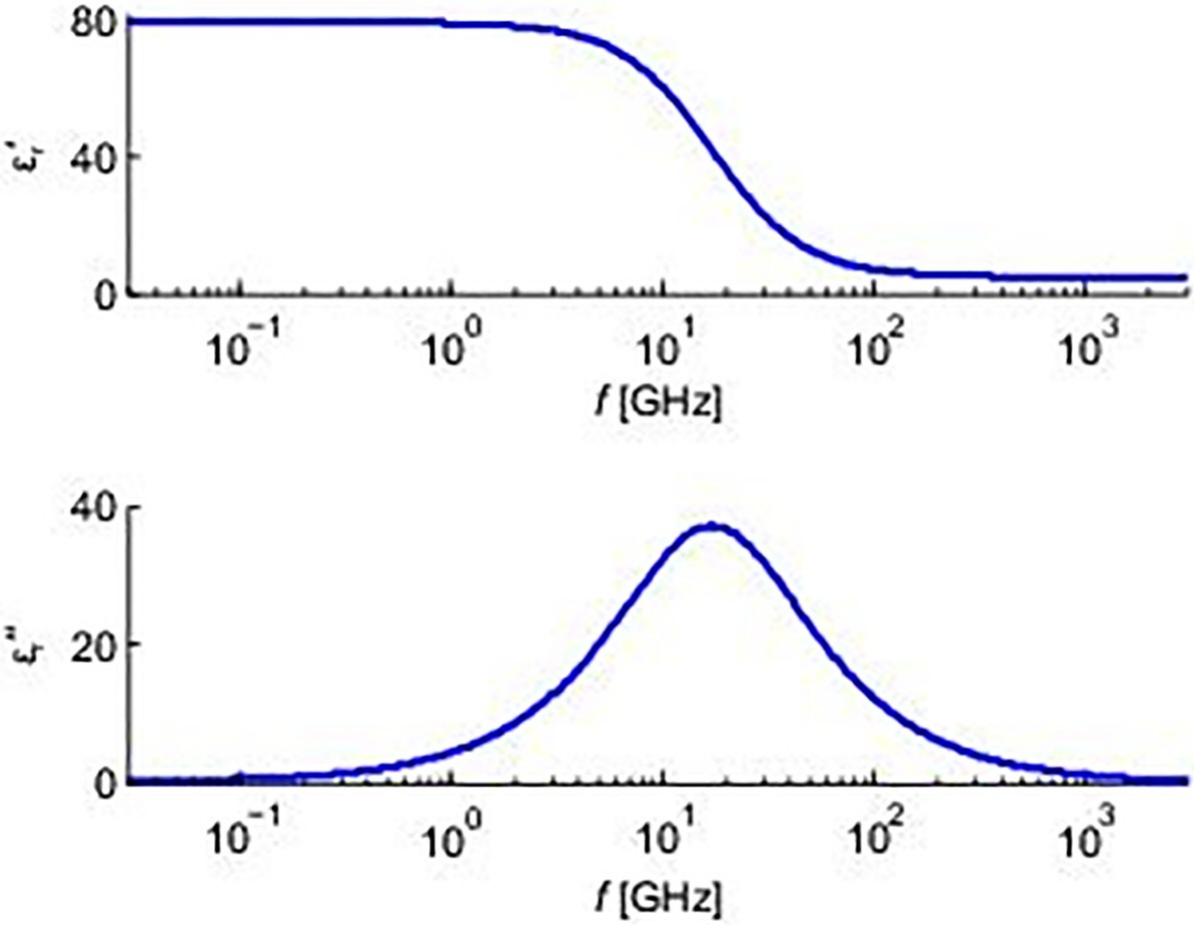
Electrical parameters of the cytosol. We used homogeneous electrical properties of the surroundings of the MTs in our model. The figure shows frequency versus complex permittivity plot. The real part of the complex permittivity (up) represents the value of the relative electrical permittivity, and therefore energy stored in the material, and the imaginary part (down) corresponds to dielectric losses.
